# Performance of physician-certified verbal autopsies: multisite validation study using clinical diagnostic gold standards

**DOI:** 10.1186/1478-7954-9-32

**Published:** 2011-08-04

**Authors:** Rafael Lozano, Alan D Lopez, Charles Atkinson, Mohsen Naghavi, Abraham D Flaxman, Christopher JL Murray

**Affiliations:** 1 Institute for Health Metrics and Evaluation, University of Washington, 2301 Fifth Ave, Suite 600, Seattle, WA 98121, USA; 2 University of Queensland, School of Population Health, Brisbane, Australia

**Keywords:** Verbal autopsy, cause of death certification, validation, physician review

## Abstract

**Background:**

Physician review of a verbal autopsy (VA) and completion of a death certificate remains the most widely used approach for VA analysis. This study provides new evidence about the performance of physician-certified verbal autopsy (PCVA) using defined clinical diagnostic criteria as a gold standard for a multisite sample of 12,542 VAs. The study was also designed to analyze issues related to PCVA, such as the impact of a second physician reader on the cause of death assigned, the variation in performance with and without household recall of health care experience (HCE), and the importance of local information for physicians reading VAs.

**Methods:**

The certification was performed by 24 physicians. The assignment of VA was random and blinded. Each VA was certified by one physician. Half of the VAs were reviewed by a different physician with household recall of health care experience included. The completed death certificate was processed for automated ICD-10 coding of the underlying cause of death. PCVA was compared to gold standard cause of death assignment based on strictly defined clinical diagnostic criteria that are part of the Population Health Metrics Research Consortium (PHMRC) gold standard verbal autopsy study.

**Results:**

For individual cause assignment, the overall chance-corrected concordance for PCVA against the gold standard cause of death is less than 50%, with substantial variability by cause and physician. Physicians assign the correct cause around 30% of the time without HCE, and addition of HCE improves performance in adults to 45% and slightly higher in children to 48%. Physicians estimate cause-specific mortality fractions (CSMFs) with considerable error for adults, children, and neonates. Only for neonates for a cause list of six causes with HCE is accuracy above 0.7. In all three age groups, CSMF accuracy improves when household recall of health care experience is available.

**Conclusions:**

Results show that physician coding for cause of death assignment may not be as robust as previously thought. The time and cost required to initially collect the verbal autopsies must be considered in addition to the analysis, as well as the impact of diverting physicians from servicing immediate health needs in a population to review VAs. All of these considerations highlight the importance and urgency of developing better methods to more reliably analyze past and future verbal autopsies to obtain the highest quality mortality data from populations without reliable death certification.

## Background

Verbal autopsy (VA) is widely used in research studies, demographic surveillance sites, and population monitoring systems [1-6]. While alternative approaches such as InterVA, the Symptom Pattern Method, and direct estimation of cause-specific mortality fractions (CSMFs) [7-13] have been used, physician review of a verbal autopsy and completion of a death certificate remains the most widely used approach for VA analysis. Physician review of VAs is based on the premise that a physician assigned the task in a given setting can correctly interpret reported signs and symptoms and occasionally household recall of health care experience (HCE) to accurately assign causes of death. Validation studies comparing physician-certified verbal autopsy (PCVA) to hospital records have shown mixed results [14-21]. The fraction of deaths where the true cause is accurately predicted has varied from 0% to 95% for different causes in these studies.

PCVA can be implemented in many different ways. Some studies or population sites use the World Health Organization-recommended VA instrument [22,23] while other sites use much more abbreviated approaches with more or less emphasis on the open or free-text component of an instrument [24,25]. PCVA also varies in the degree to which physicians undertaking VA review are trained and the curriculum of the training. Operationalization differs by the number of physicians reading each VA, the methods used to adjudicate when different physicians disagree, and the procedures to map International Classification of Diseases (ICD) codes to the physician-assigned underlying cause of death [26,27]. Interpreting the available validation studies is complicated by the considerable heterogeneity across studies in these various dimensions [28,29].

Many of the existing validation studies have several other limitations. First, in principle, validation studies compare a physician-assigned cause of death to a gold standard cause of death. But all published validation studies to date have used some form of hospital-assigned cause of death or chart review of deaths in hospital as the gold standard [30]. The quality of hospital records is highly variable, as is the underlying quality of clinical diagnosis by physicians given differences in the availability of laboratory, imaging, and pathology services. The lack of clear gold standards means that validation studies are effectively a comparison of two imperfect assignments of cause of death, not a real assessment of criterion validity. Second, by design, VA validation studies analyze deaths that occurred in a hospital or had hospital visits just prior to death. Household recall of the health care experience, including whether health workers provided documentation for the cause of hospitalization or cause of death, is part of most VA instruments. Studies in China have already shown that physician readers of VA are strongly influenced by this household recall of health care experience [11]. When health care experience recall is included in the validation studies, performance will be exaggerated when compared to how the VA will perform in populations with little or reduced access to health care. Finally, different VA validation studies have reported a wide range of metrics of validity including cause-specific sensitivity, specificity, concordance, Cohen's kappa, absolute CSMF errors, and relative CSMF errors, further complicating comparisons of performance [21,24,31,32].

The Population Health Metrics Research Consortium (PHMRC) has undertaken a five-year study to develop a range of new analytical methods for verbal autopsy and test these methods using data collected at six sites in four countries (Mexico, Tanzania, India, and the Philippines) [33]. The PHMRC study is unique both in terms of the size of the validation dataset (12,542 deaths in neonates, children, and adults) and the use of rigorously defined clinical diagnostic criteria for a death to be included in the study as a gold standard cause of death. The study was also designed to provide new evidence on issues related to PCVA, such as the impact of a second physician reader on the cause of death assigned, the variation in performance with and without household recall of health care experience, and the importance of local prior information for physicians reading VAs.

## Methods

### Gold standard cause of death assignment

The design, implementation, and general descriptive results for the PHMRC gold standard verbal autopsy validation study are described elsewhere [33]. Of note for this study, gold standard cause of death assignment was based on strict clinical diagnostic criteria defined prior to data collection. The study protocol defined three levels of cause of death assignment based on the diagnostic documentation: level 1, 2A, and 2B. Level 1 diagnoses are the highest level of diagnostic certainty possible for that condition, consisting of either an appropriate laboratory test or X-ray with positive findings, as well as medically observed and documented illness signs. Level 2A diagnoses are of moderate certainty, consisting of medically observed and documented illness signs. Level 2B was used in place of level 2A if medically observed and documented illness signs were not available, but records existed for treatment of a particular condition. Level 1 criteria were intended for all gold standard cases, and only if it proved impossible to gather enough cases of a particular condition was it allowable to use the level 2A or 2B definition. In addition to specific causes included in the list, residual categories include deaths that occur from other causes, clustered according to Global Burden of Disease categories to allow for a balanced distribution of residual causes in the data [34]. For the analysis in this paper, we present results pooling both level 1 and level 2 gold standard causes of death. Additional file 1 provides the number of adult, child, and neonatal deaths by cause used for the comparative analyses reported in this paper.

### Organization of physician review of VAs

**Figure 1:**
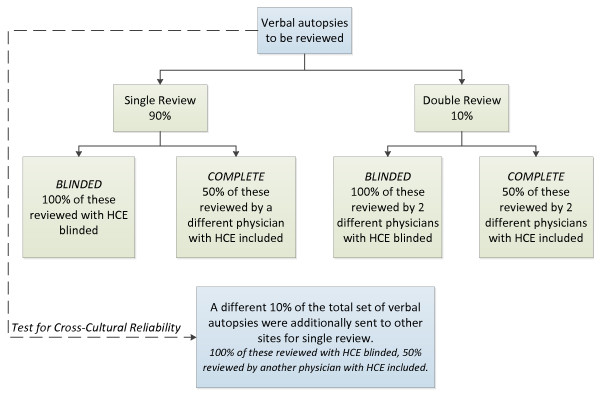
**Illustration of the review framework used for physician certification**.

Physician reviews of VAs were organized to allow testing of multiple hypotheses regarding PCVA. We wanted to evaluate the performance of PCVA in settings with and without access to health care services. To achieve this, each VA was read by a single physician, excluding items on household recall of HCE by the respondent. Half of the VAs were additionally reviewed by another physician chosen at random with household recall of health care experience included. Variables reflecting household recall of health care experience include knowledge of clinical diagnoses, records from hospital visits, death certificates, and the open-ended narrative response [33]. VAs excluding HCE are a proxy for how PCVA will perform in the community for deaths that have not occurred in a hospital or where the deceased did not have contact with the health care system. Figure 1 illustrates this review process.

To assess whether having two readers changes the performance of VA, 10% of VAs (5% with HCE) were chosen at random within each cause for review by a second physician at the same site. When the two physicians assigned different causes of death, the VA was sent to a third reader. If all three physicians disagreed, the death was assigned as indeterminate. In this paper, we do not present the results of this substudy but note that second and third review did not improve performance and in some cases made performance worse. To assess the impact of local knowledge on reading VAs, an additional 10% of VAs (5% with HCE) were assigned to a different physician from another site in another country.

Physicians in four sites were recruited to read VAs. The 24 physicians were active practitioners, English-speaking, and computer-literate. A three-day training course was organized and conducted by an experienced VA analyst to provide all physicians with a similar basis for their work. The training curriculum was based on a customized version of the Sample Vital Registration with Verbal Autopsy (SAVVY) manual [35]. VAs were randomly assigned to physicians. Household recall of health care experience and records were identified as direct diagnosis questions, medical records, death certificates, and open-ended responses. For reviews excluding these items, physicians were shown a PDF of the VA instrument without this information provided. For the 10% of VAs sent to another country, the open-ended material and information from the death certificate was first translated into English.

**Figure 2:**
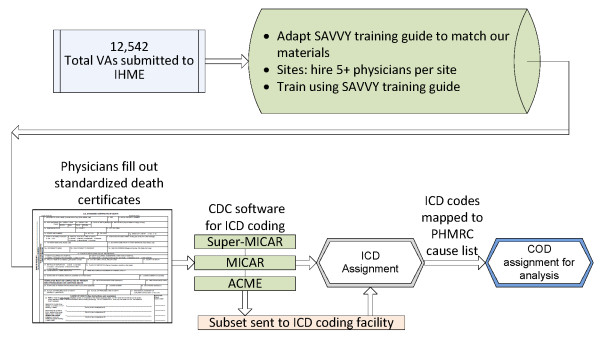
**Diagram of the process for physician review and data analysis**.

For each VA, the physician would read the instrument and complete a WHO standard death certificate. The completed death certificate was processed through the US Centers for Disease Control and Prevention's Mortality Medical Data System (MMDS) software [36] for automated ICD-10 coding of the underlying cause of death. Approximately 25% of certificates were rejected by the MMDS software. These rejected certificates were sent to the National Institute of Health Sciences in Sri Lanka for manual ICD-10 coding. The ICD-10 codes were then mapped to the PHMRC cause list to allow for direct comparison to the gold standard. Figure 2 summarizes the physician review process.

### Data analysis

We have analyzed the performance of physician review using the metrics recommended by Murray et al. (2011) [37]. The analyses for neonates, children, and adults were conducted separately. The numbers of causes including residual causes of death were 34 causes for adults, 21 for children, and six for neonates. The reasons behind the decision to reduce the number of causes from the original design are explained in detail elsewhere [33]. In the case of neonates and specifically for PCVA analysis, the cause list had to be reduced to five causes of death plus stillbirths. This is because the set of causes included for the validation study of combinations of prematurity with various other conditions do not have unique ICD codes in the 10^th ^revision [38]. For this study, underlying cause of death was assigned following the rules of the ICD for each sequence of causes of death that the physicians produced after reading the VA. For example, we aggregated in preterm delivery all deaths from five causes from the original list, such as preterm delivery without respiratory distress syndrome (RDS), preterm delivery (without RDS) and birth asphyxia, preterm delivery (with or without RDS) and sepsis, preterm delivery (without RDS) and sepsis/birth asphyxia, and preterm delivery with RDS. These more refined causes of death for neonates reflect the presence of comorbid conditions; while they have clear relevance to understanding patterns of neonatal mortality, they do not map to the ICD-10.

To compute the median chance-corrected concordance and CSMF accuracy for each category, we first created 500 test datasets with true CSMF compositions drawn from an uninformative Dirichlet distribution for the relevant number of causes by sampling within each cause with replacement. For each draw, we compute chance-corrected concordance and CSMF accuracy and report the median value across the draws. We also calculated a linear regression of true and estimated CSMFs for each cause. The slope and intercept measure how accurately the estimated cause matches the true cause, with a slope of 1 and intercept of 0 indicating a perfect match. The root mean square error (RMSE) indicates how precisely the cause is estimated, with lower RMSE values indicating greater correlation.

We used random effects logistic regression to study the factors associated with physicians assigning the true cause to a death. Independent variables included fixed effects for level of gold standard diagnosis, whether the VA was reviewed at the site it was collected or a different site, and inclusion of information on the household recall of health care experience, as well as random effects for cause and physician nested by site. We also conducted a sensitivity analysis to determine if physicians assigned the correct cause of death in any of the diagnoses from the death certificate rather than as just the underlying cause itself.

## Results

### Individual cause assignment

Table 1 shows the overall results for the performance of PCVA against the gold standard cause of death. Without household recall of health care experience, a proxy for PCVA in communities with limited access, physicians get the cause right after correcting for chance less than 30% of the time in adults and neonates, and 36% of the time in children. Providing physicians with items on health care experience and the free-text components improves performance markedly in adults to 45% and slightly higher in children to 48%. Despite the short cause list in neonates, chance-corrected concordance only increases to 33%. In all cases, PCVA has chance-corrected concordances of less than 50%.

**Table 1:** Median chance-corrected concordance (%) and 95% uncertainty interval [UI], by age group with and without HCE

	No HCE		HCE	
	Median	95% UI	Median	95% UI
**Adults**	29.7	(29.4, 29.8)	44.6	(44.3, 44.8)
**Children**	36.3	(35.9, 36.6)	47.8	(47.1, 48.3)
**Neonates**	27.6	(27.2, 28.0)	33.3	(32.8, 33.7)

**Figure 3:**
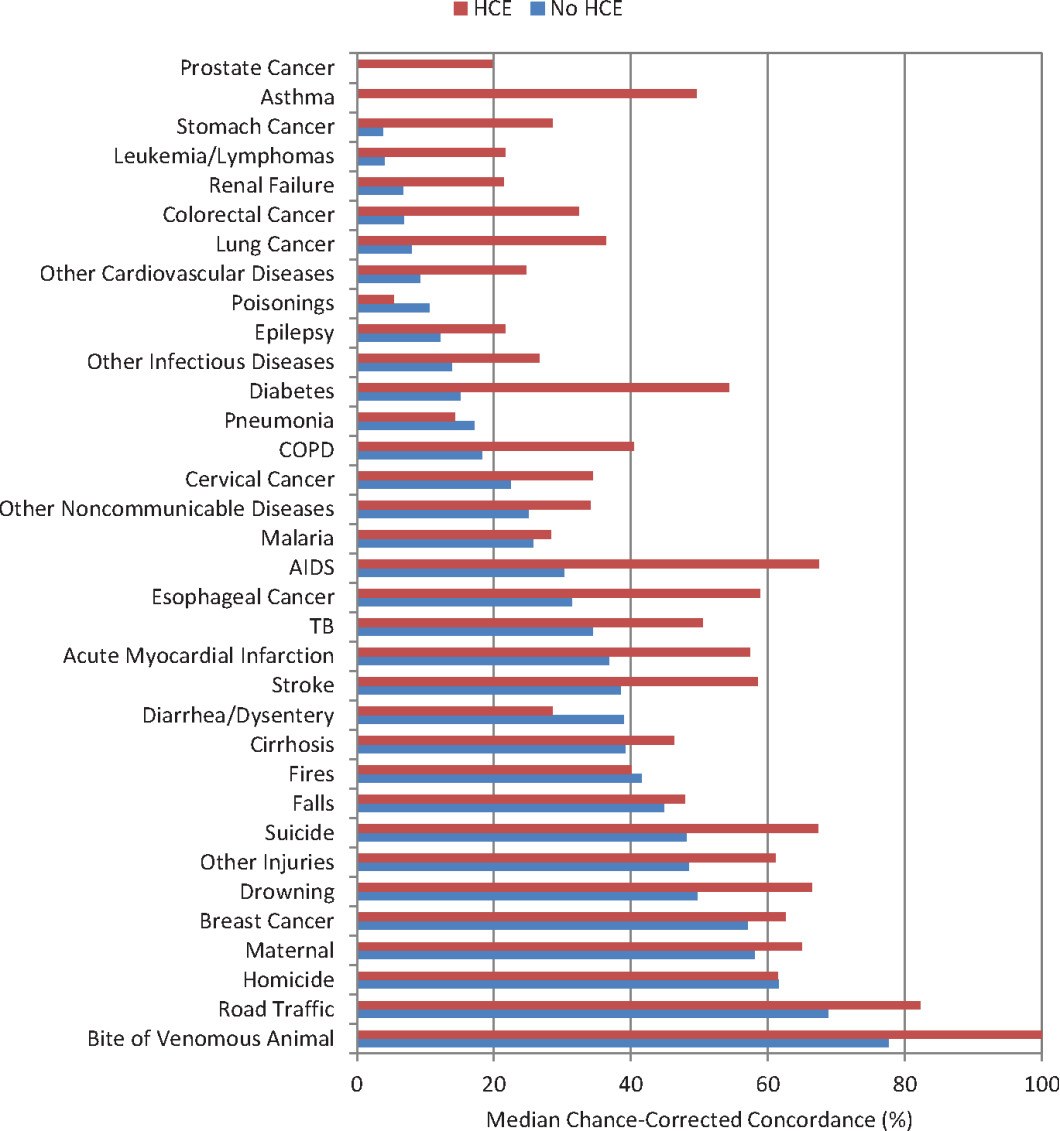
**Median chance-corrected concordance (%), by adult cause with and without HCE**.

**Figure 4:**
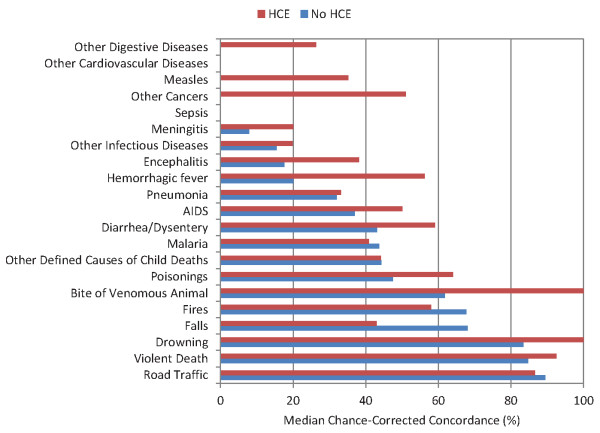
**Median chance-corrected concordance (%), by child cause with and without HCE**.

**Figure 5:**
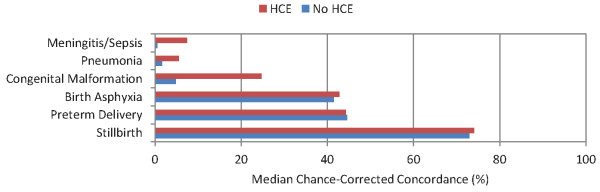
**Median chance-corrected concordance (%), by neonate cause with and without HCE**.

Chance-corrected concordance by cause with and without HCE is shown in Figure 3 for adults, Figure 4 for children, and Figure 5 for neonates; detailed values and uncertainty intervals are provided in Additional file 2. Physicians are able to achieve a chance-corrected concordance of 50% or greater in adults for a number of injuries (bite of a venomous animal, road traffic accidents, homicides, drowning), maternal causes, and breast cancer. When HCE is included in the VA, chance-corrected concordance increases enough so that other injuries, suicides, AIDS, acute myocardial infarction, and stroke cross the 50% threshold. Of note, PCVA does extremely poorly for some important causes of death such as prostate cancer, stomach cancer, leukemia/lymphoma, epilepsy, renal failure, colorectal cancer, poisonings, diabetes, asthma, and pneumonia. Addition of HCE notably improves performance for asthma and diabetes in this grouping.

The same analysis in children shows that physician review does well for a number of injuries including violence, road traffic, drowning, fires, falls, and bite of a venomous animal. Falls is one case where addition of the health care experience information actually lowers chance-corrected concordance. Some major causes of death such as diarrhea/dysentery, malaria, and AIDS have intermediate levels of performance. On the other hand, pneumonia has a chance-corrected concordance below 33% with and without HCE. Somewhat surprisingly, PCVA has quite poor performance for the limited number of measles deaths in the study. Physicians do not perform better than or worse than chance for some causes such as sepsis, other cardiovascular diseases, and other digestive diseases.

For the neonatal death analysis examining only a five-cause list and stillbirths, PCVA achieves chance-corrected concordance greater than 50% only for stillbirths. Chance-corrected concordance is intermediate in value for birth asphyxia and preterm delivery but very poor for congenital malformation, pneumonia, and meningitis/sepsis.

Table 2 reports on the determinants of concordance using mixed-effects logistic regression. The regression controls for cause (coefficients not shown) and site/physician, and includes independent variables for the availability of HCE, whether the review was in-site or out-of-site, and a dummy variable indicating whether the death met only level 2 gold standard criteria. Table 2 confirms the overall finding that availability of HCE makes a profound difference in the probability that a physician will assign the true cause as the underlying cause of death. The odds ratio is highest in adults and much lower in neonates, indicating that there is perhaps more useful information in health care experience for assigning adult causes than for neonates and children. For all age groups, physicians performed slightly better reviewing in-site VAs, suggesting that prior knowledge of causes of death and associated symptoms may influence their concordance, with the greatest effect in children. In adults, physicians are less likely to get the true cause correct when the diagnostic criteria only meet level 2, but the reverse is true in children. This may be explained by the fact that the same clinical history used in the absence of laboratory confirmation for some level 2 diagnoses in children are what physicians use to assign cause in a VA.

**Table 2:** Mixed-effects logistic regression odds ratios (OR) and standard errors (SE), by determinant of concordance

	Adult		Child		Neonate	
	OR	SE	OR	SE	OR	SE
**With HCE**	2.03	0.08	1.38	0.11	1.11	0.08
**In-site**	1.22	0.10	1.71	0.28	1.29	0.16
**Gold Standard Level 2**	0.87	0.06	1.36	0.16	1.61	0.85

**Figure 6:**
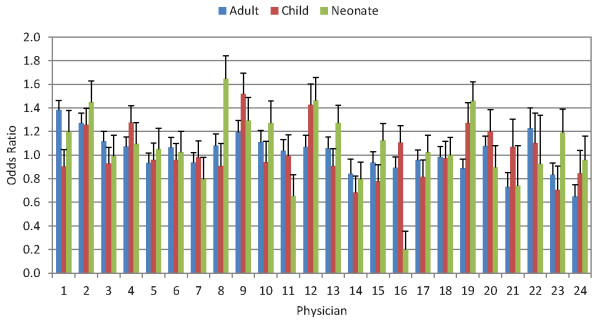
**Random effect logistic regression odds ratios (OR) and standard errors (SE) by physician, of assigning the correct cause as a function of the physician reading the VA for adult, child, and neonatal causes**.

Figure 6 shows the odds ratio of assigning the correct cause as a function of the physician reading the VA for adult, child, and neonatal causes. For adult causes, the odds ratio for getting the true cause correct ranges from 0.65 to 1.43. For children, there is a similarly wide range across physicians and an even broader variation in performance across physicians for neonates. One physician, for example, has an odds ratio of 0.20 for neonates. This analysis demonstrates that after controlling for cause and information available on the VA, there is substantial variation in physician performance. We cannot determine the attributes of success but they most likely include training, clinical experience, and diagnostic skill.

### CSMF estimation

The overall accuracy of physicians in estimating CSMFs for the test set is given in Table 3. CSMF accuracy across 500 test sets shows that physicians estimate CSMFs with considerable error for adults, children, and neonates. Only for neonates with HCE is accuracy above 0.7. In all three age groups, CSMF accuracy improves when household recall of health care experience is available.

**Table 3:** Median CSMF accuracy and 95% UI, by age group with and without HCE

	No HCE		HCE	
	Median	95% UI	Median	95% UI
**Adults**	0.624	(0.619, 0.631)	0.675	(0.669, 0.680)
**Children**	0.632	(0.626, 0.642)	0.682	(0.671, 0.690)
**Neonates**	0.695	(0.682, 0.705)	0.733	(0.719, 0.743)

**Figure 7:**
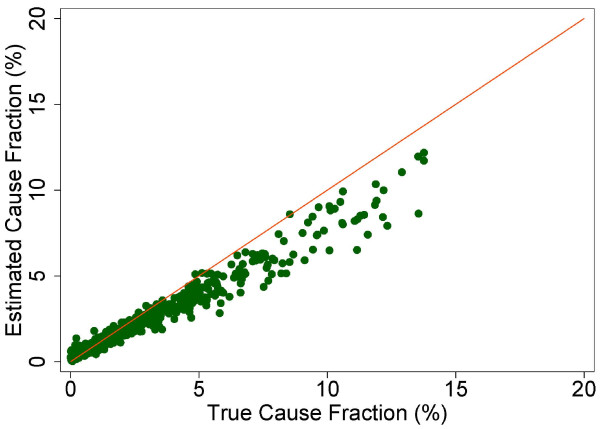
**Estimated versus true CSMFs across 500 Dirichlet splits, for adult bite of venomous animal without HCE**.

**Figure 8:**
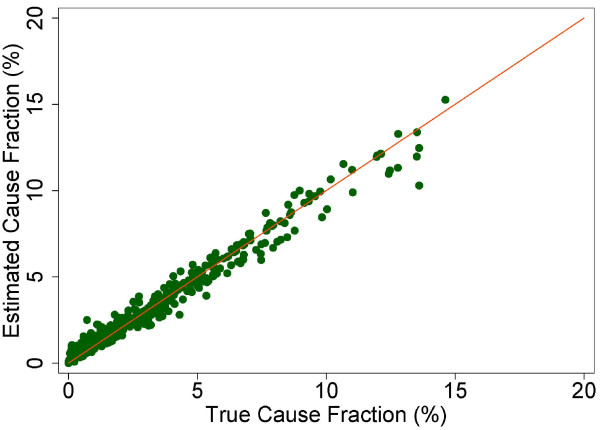
**Estimated versus true CSMFs across 500 Dirichlet splits, for adult bite of venomous animal with HCE**.

**Figure 9:**
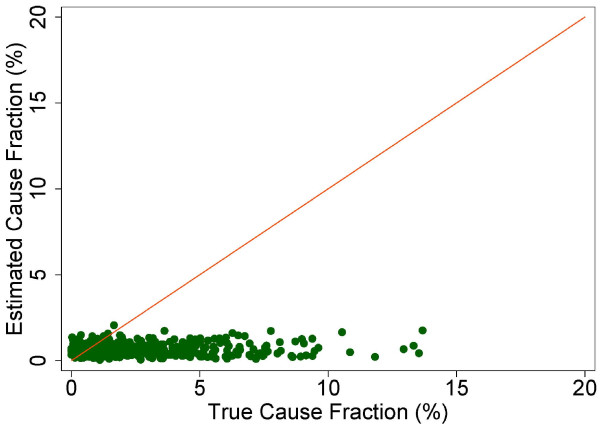
**Estimated versus true CSMFs across 500 Dirichlet splits, for adult asthma without HCE**.

**Figure 10:**
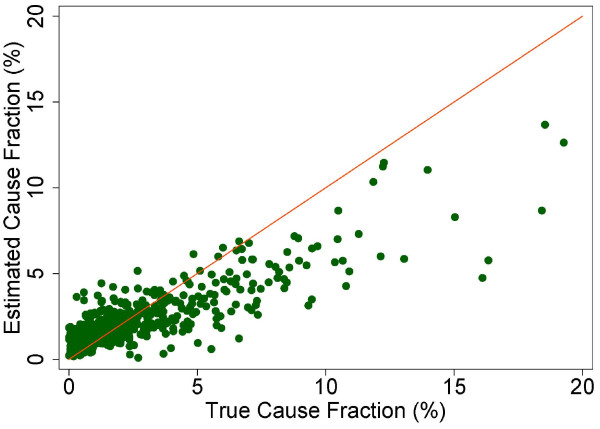
**Estimated versus true CSMFs across 500 Dirichlet splits, for adult asthma with HCE**.

**Figure 11:**
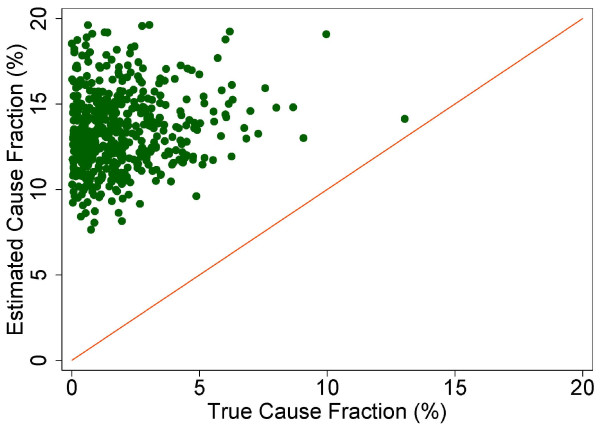
**Estimated versus true CSMFs across 500 Dirichlet splits, for adult other noncommunicable diseases without HCE**.

**Figure 12:**
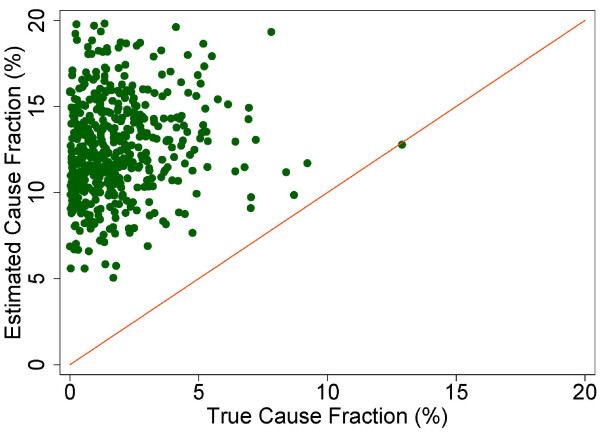
**Estimated versus true CSMFs across 500 Dirichlet splits, for adult other noncommunicable diseases with HCE**.

**Figure 13:**
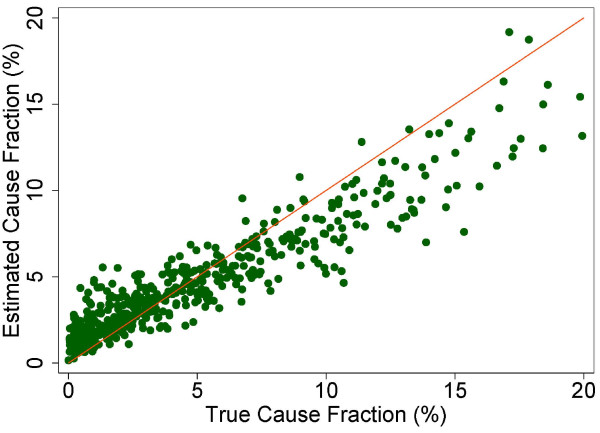
**Estimated versus true CSMFs across 500 Dirichlet splits, for child falls without HCE**.

**Figure 14:**
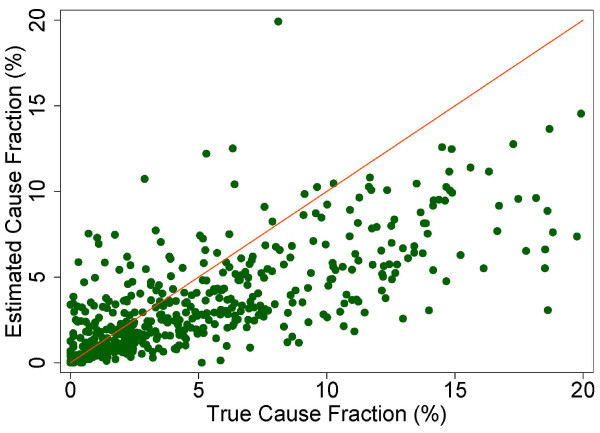
**Estimated versus true CSMFs across 500 Dirichlet splits, for child falls with HCE**.

A more fine-grained appreciation of how well PCVA does in estimating CSMFs is provided in Figure 7 for adult bite of a venomous animal without HCE and Figure 8 for adult bite of a venomous animal with HCE, Figure 9 for adult asthma without HCE and Figure 10 for adult asthma with HCE, Figure 11 for adult other noncommunicable diseases without HCE and Figure 12 for adult other noncommunicable diseases with HCE, and Figure 13 for child falls without HCE and Figure 14 for child falls with HCE. For selected causes with and without HCE, CSMFs as estimated through PCVA are compared to the true CSMFs in the test datasets. Figure 7 and 8 show that with or without HCE, PCVA does a reasonably good job estimating the cause fraction due to bite of a venomous animal. Even in this case, inclusion of the HCE, especially the open-ended narrative, improves CSMF estimation. Figure 9 shows that for asthma without HCE, estimated CSMFs are almost always too low and do not tend to be higher when the true CSMF is higher. In contrast, adding HCE to the VA (Figure 10) yields CSMF estimates that are too high at low true CSMFs and too low at high true CSMFs. Figures 11 and 12 illustrate a systematic problem with PCVA: the tendency to assign to the residual category of other noncommunicable diseases far too many deaths. In fact, in nearly every case, the estimated CSMF is substantially higher than the true CSMF. Further, there is no correlation between the estimated and true CSMFs. Where PCVA says there are more deaths from other noncommunicable diseases compared to another population, this relationship implies there may not be more deaths in reality. Figures 13 and 14 show that, for child falls, addition of HCE actually causes both overestimation and underestimation to increase when the true CSMF is higher.

Additional file 3 shows the slope, intercept, and RMSE results from the linear regression by cause. As expected, causes with accurate estimation (injuries, breast cancer, maternal, stillbirths) have a slope near 1 and intercept near 0, while causes with inaccurate estimation (sepsis, meningitis, pneumonia, asthma, and the other residual categories) have a lower slope and higher intercept. Similarly, high-correlation causes (injuries, cancers, stillbirths) have a low RMSE, and low-correlation causes (pneumonia, malaria, diarrhea/dysentery, birth asphyxia, and other residual categories) have a high RMSE. Some causes have accurate estimation and low correlation (homicide, violent death) while other causes have inaccurate estimation and high correlation (cancers, epilepsy, asthma). Physicians are better overall at estimating CSMFs for adults than for children and neonates. For nearly all causes, addition of HCE leads to more accurate CSMF estimation. Notable exceptions are diarrhea/dysentery in adults and falls in children, for which we observed a similar decrease in chance-corrected concordance. Interestingly, addition of HCE decreases the correlation of CSMF estimation for most causes, most substantially for asthma and diabetes in adults, other infectious diseases and poisonings in children, and congenital malformation and meningitis/sepsis in neonates.

### Coding sensitivity

In the study protocol, following recommendations from the WHO, the physician reading the VA completes a death certificate. The final underlying cause assigned is based on processing this death certificate using MMDS software or manual coding for those rejected by the software. We studied the extent to which the physician may be assigning the true cause of death on the death certificate in one of the additional cause lines as opposed to the underlying cause, or where the other causes assigned combined with ICD rules leads to the assignment of an underlying cause that is different from the gold standard cause of death. We tested this by calculating the partial chance-corrected concordance, assigning a physician as concordant if s/he assigns the true cause of death in any of the lines of the death certificate. Partial chance-corrected concordance takes into account that, automatically by chance, physicians would assign the true cause in either the underlying or associated causes of death more often. Table 4 shows that the partial chance-corrected concordance increases in reviews without HCE in adults and children by 2.1% and 1.2% respectively. In neonates, the partial chance-corrected concordance actually declines by 2.9%. With HCE, the change is more substantial, 4.5% and 2.3% in adults and children respectively. For neonates, as without HCE, it declines, this time by 4.6%.

**Table 4:** Sensitivity analysis comparing partial chance-corrected concordance (%) for correct cause assignment with underlying versus all diagnoses

	Underlying		All Diagnoses	
	No HCE	HCE	No HCE	HCE
**Adults**	29.7	44.6	31.8	49.1
**Children**	36.3	47.8	37.5	50.1
**Neonates**	27.6	33.3	24.7	28.7

## Discussion

When physicians review VA results for individuals who died without contact with health care services, the median chance-corrected concordance ranges from -3% to 77.6% with an average value across causes of 29.7% for adults; -5% to 89.5% with an average value of 36.3% for children; and 1.6% to 72.9% with an average value of 27.6% for neonates. This basic result is the same whether one or two physicians review the VA but is lower when physicians from other locations review the VA. Performance improves when physicians are given access to household recall of health care experience and medical records retained by the household. Both results, the improvement with HCE and the difference between physicians from within the country versus physicians from another country, highlight that a substantial component of VA diagnoses are a function not of signs and symptoms but the combination of prior epidemiological views of the physician reader and filtered information on medical records provided by the household. In other words, the validity of PCVA is highly contextual. It will perform better when respondents have more access to health care and when physicians are strongly guided by their prior beliefs on the prevalence of diseases.

Performance of a VA method on estimating CSMFs is a complex function of both individual death assignment concordance and the pattern of how true negatives are larger or smaller than false positives. The median CSMF accuracy found in this study was 0.624 without HCE and 0.675 with HCE for adults; 0.632 without HCE and 0.682 with HCE for children; and 0.695 without HCE and 0.733 with HCE for neonates. The performance of PCVA must be interpreted in light of the performance of medical certification of causes of death in a functioning vital registration system. Hernández et al. (2011) [39] have found in Mexico, for example, that routine medical certification using the same gold standard deaths has a median chance-corrected concordance of 66.5% for adults, 38.5% for children, and 54.3% for neonates; and a CSMF accuracy of 0.780 for adults, 0.683 for children, and 0.756 for neonates. This is one of the few studies with comparable assessment of medical certification of death using the same methods and metrics. PCVA provides less accurate measurement than medical certification for adults but comparable results for children and neonates.

To many readers, the relatively modest performance of PCVA will come as a surprise. Some previously published studies [14-20] have reported substantially higher concordances compared to medical record review and quite small errors in estimated CSMFs. The less impressive performance reported here must be viewed taking into account two factors. First, in this study PCVA is being compared to a true gold standard. It is possible that the same signs and symptoms that lead to diagnoses in some facilities without laboratory tests or diagnostic imaging are those used by physicians reading a VA leading to falsely inflated performance when no gold standard is available. Second, by assessing PCVA performance estimating CSMFs across 500 test datasets, we get a much more robust assessment of performance at estimating CSMF performance, an assessment that is not simply the function of the CSMF composition in one particular test dataset.

The findings on PCVA must also be interpreted in light of the results of the sensitivity analysis. In the adult case with HCE, in 5% of the deaths, physicians assign the true cause somewhere on the death certificate but not as underlying cause. Our study is a fair assessment of the cause of death pattern yielded through PCVA using a rigorous protocol for coding causes of death. The sensitivity result, however, suggests that better training of physicians in completing the death certificate might improve performance. In this study, physicians were carefully trained in this part of the completion of a VA. The difference for children and neonates is less marked. In addition to the discrepancy in coding sensitivity, several of the physicians experienced difficulty in completing their assigned VAs due to the length of time involved in reading each VA. In some cases, VAs had to be reassigned to a different physician at the same site to ensure completion. The results of this study were conducted with 95% of the total VAs sent out for review.

We present results based on a single physician review of each VA. We have as part of this broader study a substudy comparing single review and double review with adjudication of conflicting reviews. For reasons of space, we have not presented the results from that substudy here. Our overall conclusions, however, presented in this paper on PCVA will not be affected by using only single review. In fact, we find that two readers do not improve performance over a single reader, confirming a result published for Andhra Pradesh [40]. Based on purely probability theory grounds, double review should only improve the results of VA if a single physician is more than 50% likely to get the true cause correct. Given that a single physician is less than 50% likely to get the true cause correct, there is no theoretical argument in favor of double review, nor is there empirical support in our study.

Our finding that physicians vary markedly in their ability to assign the true cause controlling for cause of death, availability of HCE, and whether a physician is from the site or another location has important implications. It suggests that despite standardized training, all physicians are not equal in their ability to assign causes of death. Given that physicians vary in diagnostic skill for patients when they are alive, it should not be surprising that some physicians are better than others at reading verbal autopsies. This reality is one further challenge to implementing PCVA. The marked sensitivity of the results to the diagnostic ability of different physicians and their prior views on the prevalence of diseases suggests that more rigorous screening and training of physicians who undertake PCVA could improve the results. This highlights the major implementation challenge that many are facing: it is costly, time-consuming, and difficult to recruit and motivate physicians to read large numbers of VAs. Recruiting physicians with better diagnostic acumen and ability to accurately assign causes of death given a VA could be even more problematic. PCVA by its nature has substantially lower reproducibility than automated statistical or machine-learning methods for VA analysis.

## Conclusions

Given the cost, implementation difficulty, and idiosyncratic nature of PCVA, what should be its role in future VA data analysis? Clearly, more rigorous standardization of questionnaire implementation, tests of diagnostic skill, and training might be able to improve concordance and perhaps increase CSMF accuracy. These efforts will likely increase costs and delays in implementation. If lower-cost, more-reproducible methods can perform as well as PCVA, they would have substantial advantages for many data-collection platforms. The challenge for physicians to assign an accurate cause of death on the basis of the recall of signs, symptoms, and health care experience raises questions about the accuracy of medical certification of deaths that occur outside of a health facility. In many countries, medical certification of these deaths has the same or a more limited information basis available for the physician completing the death certificate. If alternative methods for assigning verbal autopsy causes of death are available, they may have an important role in medical certification of death outside of health facilities.

To our knowledge, this is the first true validation study where the performance of PCVA has been compared to a rigorously defined gold standard cause of death. Given that verbal autopsy remains the global standard for assessing causes of death and prioritizing health interventions in areas lacking complete vital registration systems, it is essential to develop analytical methods that are low-cost, quick to implement, and consistently accurate. Physician review meets none of these criteria, and yet it is still the most widely implemented method for analysis of VAs today. As a result, verbal autopsy studies that rely on physician coding for cause of death assignment may not be as robust as previously thought. The time and cost required to initially collect the verbal autopsies must be considered in addition to the analysis, as well as the impact of diverting physicians from servicing immediate health needs in a population to review VAs. All of these considerations highlight the importance and urgency of developing better methods to more reliably analyze past and future verbal autopsies to obtain the highest quality mortality data from populations without reliable death certification.

## Abbreviations

CSMF: cause-specific mortality fraction; HCE: health care experience; ICD: International Classification of Diseases; MMDS: Mortality Medical Data System; PCVA: physician-certified verbal autopsy; PHMRC: Population Health Metrics Research Consortium; RMSE: root mean square error; SAVVY: Sample Vital Registration with Verbal Autopsy; VA: verbal autopsy; WHO: World Health Organization

## Competing interests

The authors declare that they have no competing interests.

## Authors' contributions

RL, ADL, ADF, and CJLM conceptualized and guided the study. CA performed analyses and helped write the manuscript. MN mapped cause lists and ICD codes. CJLM drafted the manuscript and approved the final version. CJLM accepts full responsibility for the work and the conduct of the study, had access to the data, and controlled the decision to publish. All authors have read and approved the final manuscript.

## Supplementary Material

Additional file 1 **Number of deaths for adult, child, and neonate causes in the PHMRC study**. Click here for file

Additional file 2 **Median chance-corrected concordance (%) and 95% UI, by cause with and without HCE**. Click here for file

Additional file 3 **Slope, intercept, and RMSE from linear regression of estimated versus true CSMFs, by cause with and without HCE**. Click here for file
